# The Role of Moral Injury in PTSD Among Law Enforcement Officers: A Brief Report

**DOI:** 10.3389/fpsyg.2020.00310

**Published:** 2020-03-04

**Authors:** Konstantinos Papazoglou, Daniel M. Blumberg, Victoria Briones Chiongbian, Brooke McQuerrey Tuttle, Katy Kamkar, Brian Chopko, Beth Milliard, Prashant Aukhojee, Mari Koskelainen

**Affiliations:** ^1^Yale School of Medicine, New Haven, CT, United States; ^2^California School of Professional Psychology, Alliant International University, San Diego, CA, United States; ^3^MV Stats Consultants, San Mateo, CA, United States; ^4^Center for Family Resilience, Oklahoma State University, Tulsa, OK, United States; ^5^Department of Psychiatry, University of Toronto, Toronto, ON, Canada; ^6^Centre for Addiction and Mental Health, Toronto, ON, Canada; ^7^Department of Sociology, Kent State University, North Canton, OH, United States; ^8^York Regional Police, Aurora, ON, Canada; ^9^Department of Psychology, University of Toronto, Toronto, ON, Canada; ^10^National Bureau of Investigation, Intelligence Division, Threat Assessment Team, Vantaa, Finland

**Keywords:** law enforcement, moral injury, PTSD, compassion fatigue, trauma, health, resilience

## Abstract

Exposure to critical incidents and hence potentially traumatic events is endemic in law enforcement. The study of law enforcement officers’ experience of moral injury and their exposure to potentially morally injurious incidents, and research on moral injury’s relationship with different forms of traumatization (e.g. compassion fatigue, post-traumatic stress disorder) are in their infancy. The present study aims to build on prior research and explores the role of moral injury in predicting post-traumatic stress disorder (PTSD) and its clusters thereof. To this end, a sample of law enforcement officers (*N* = 370) from the National Police of Finland was recruited to participate in the current study. Results showed that moral injury significantly predicted PTSD as well as its diagnostic clusters (i.e., avoidance, hyperarousal, re-experiencing). The aforementioned role of moral injury to significantly predict PTSD and its clusters were unequivocal even when compassion fatigue was incorporated into the path model. Clinical, research, and law enforcement practice implications are discussed.

## Introduction

Moral injury has received burgeoning attention in the scholarly literature. In fact, a recent special issue of *Journal of Traumatic Stress* was dedicated to a discussion of moral injury and included a comprehensive review of the literature on this topic (Griffin et al., 2019). According to the U.S. Department of Veterans Affairs, National Center for PTSD, moral injury refers to unprecedented traumatic life events wherein one perpetrates, fails to prevent, or witnesses actions that “transgress deeply held moral beliefs and expectations” (Litz et al., 2009, p. 1). Thus, the key precondition for moral injury is an act of transgression, which shatters moral and ethical expectations that are rooted in religious or spiritual beliefs, or culture-based, organizational, and group-based rules about fairness, the value of life, and so forth. Conceptually, attention has focused on examining outcomes associated with moral injury (e.g. Litz and Kerig, 2019; Nash, 2019), defining potentially morally injurious events (PMIEs; e.g. Yeterian et al., 2019), and differentiating both index events and outcomes associated with moral injury and post-traumatic stress disorder (PTSD; e.g. Jinkerson, 2016; Currier et al., 2019). Practically, questions remain regarding best practices for treating moral injury and the extent to which current treatments for PTSD are suitable to treat moral injury (e.g. Barnes et al., 2019; Griffin et al., 2019; Neria and Pickover, 2019) or if new treatments designed specifically for moral injury would be more efficacious (e.g. Nash and Litz, 2013; Jordan et al., 2017).

Researchers have examined the relationship between moral injury and PTSD. Although the two conditions can stem from similar events (when meeting DSM-5 Criterion A for PTSD), many PMIEs do not meet diagnostic criteria for PTSD. Jordan et al. (2017) differentiated perpetration-based PMIEs from betrayal-based PMIEs. They found that guilt/shame mediated PTSD symptoms related to perpetration PMIEs, whereas anger mediated the PTSD symptoms related to betrayal PMIEs (p. 632). Regardless of the precipitant, however, the outcomes appear to share many characteristics, and the two conditions have been observed to co-occur among military samples (e.g. Jordan et al., 2017; Currier et al., 2019; Davies et al., 2019). In one study, higher levels of moral injury correlated positively with “increased history of suicide attempt, but only among those higher in PTSD severity” (Bryan et al., 2018, p. 41). In another recent study, moral injury scores correlated with “worse symptom severity of PTSD and major depressive disorder across the 6-month period” (Currier et al., 2019, p. 388). These researchers also found that “only self-directed moral injury emerged as a salient predictor of PTSD symptoms in the primary analysis” (Currier et al., 2019, p. 390). Nevertheless, research has confirmed that PTSD and moral injury are separate constructs (Bryan et al., 2018). Moreover, neurobiological studies using fMRI have demonstrated that “moral injury, while often coexistent with PTSD, is not fear-based” (Barnes et al., 2019, p. 101).

Despite the increased focus on the construct, the empirical study of moral injury has centered almost exclusively on samples of active military personnel and veterans. Recent work explored aspects of moral injury with social workers (Haight et al., 2016), with journalists (Feinstein et al., 2018), on civilian populations (Steinmetz et al., 2019) and with emerging adults (Chaplo et al., 2019). Police officers are another population impacted by moral injury. Police officers, due to the moral risks associated with their jobs (Blumberg et al., 2018), experience PMIEs and are vulnerable to the development of morally injurious outcomes (Papazoglou et al., 2019b). One study looked at the impact on police officers who killed or injured someone (Komarovskaya et al., 2011). Although they did not directly assess moral injury, these types of perpetration-based PMIEs were correlated with PTSD and, to a lesser degree, depression, but not with social adjustment or alcohol usage (Komarovskaya et al., 2011, p. 1334). Nevertheless, there remains a paucity of attention on moral injury experienced by police officers (e.g. Blumberg and Papazoglou, 2019; Papazoglou et al., 2019a).

The purpose of this study is to examine the role of moral injury in predicting PTSD as well as its diagnostic clusters (i.e., re-experiencing, avoidance, hyperarousal). The study is the first to examine these variables among police officers; hence, it provides an important look at the role that moral injury plays in the PTSD symptomology of police officers. Even though the authors of the present study included compassion fatigue in the current data analysis to explore its role as a trauma-related condition, along with moral injury, in predicting PTSD (and its associated clusters), the main focus of the paper, as mentioned above, is on moral injury and PTSD. For those who are interested in reading further about compassion fatigue and its relationship with moral injury, personality traits, and overall police traumatization; the authors refer readers to previously published work (e.g. Papazoglou et al., 2019a; Tuttle et al., 2019).

## Methods^1^

### Participants

The study participants were 370 uniformed, operational law enforcement officers working for the Finnish national police. All research participants were white Europeans, the majority being male officers (73.5%). They averaged 16.87 (*SD* = 9.11) years of police experience, which involved working in units where they were susceptible to experiencing direct or indirect traumatic events that affected their psychological health and functioning. Descriptive and demographic statistics regarding the study sample are presented in Tables 1, 2.

**TABLE 1:** Descriptive statistics for the demographic and study variables (*N* = 370).

Variables	α	Range	*M*	*SD*
**Demographics**				
Age in years	–	23 to 62	41.21	8.42
Years in police service	–	1 to 42	16.87	9.11
Years in current job	–	0 to 34	7.98	6.66
**Study variables**				
Compassion fatigue	0.90	0.04 to 3.57	1.04	0.55
PTSD total	0.93	0.98 to 4.64	1.62	0.59
Re-experiencing (B)	0.89	1.00 to 4.20	1.43	0.60
Avoidance (C)	0.86	0.88 to 4.86	1.64	0.67
Hyperarousal (D)	0.80	0.92 to 5.00	1.80	0.69
Moral injury	0.75	1.00 to 6.00	3.34	0.86

**TABLE 2:** Unstandardized and standardized path coefficients for the PTSD cluster model (*N* = 370).

				95% CI	
Path	*B*	*SE*	β	Lower	Upper
**Compassion fatigue to:**					
Re-experiencing	0.39	0.04	0.64**	0.55	0.70
Avoidance	0.38	0.04	0.59**	0.52	0.65
Hyper-arousal	0.39	0.04	0.60**	0.52	0.66
**Moral injury to:**					
Re-experiencing	0.03	0.04	0.08**	0.01	0.16
Avoidance	0.09	0.04	0.22**	0.14	0.30
Hyper-arousal	0.08	0.04	0.18**	0.10	0.26

Bootstrap *SE* are reported. CI are bias-corrected. **p < 0.01.

### Procedures

Police officers within several national Finnish agencies (e.g. Police of Finland, National Bureau of Investigation, Police University College and the Security Intelligence Service) received a hyperlink to the survey via “Webropol,” a versatile and highly secured survey tool used by the Finnish police to gather information from their personnel. Once officers voluntarily consented to partake in the study through Webropol, they subsequently provided demographic information (e.g. years of service, type of field placement) and responded to survey items on topics ranging from their current work situation (e.g. *my work makes me feel satisfied*) to their perception of self and others based on incidents (e.g. *I am troubled by having witnessed others’ immoral acts*). Participation was voluntary and no compensation was offered. Data collection occurred during normal working hours. The Research Ethics Board of the National Police of Finland as well as the University of Toronto approved the current study before initial data collection.

### Measures

#### Demographics

Demographic measures included officers’ age, gender, field of work, number of years worked, racial background (e.g. Asian, Black, White, Other) and ethnicity (e.g. Arab, Jewish, Hispanic/Latino, Other).

#### Compassion Fatigue

To assess compassion fatigue, the *Compassion Satisfaction and Fatigue Test* (CSF – Figley and Stamm, 1996). (CSF) was used. Officers were asked to self-report their experience by answering a Likert-type scale of 66 items (e.g. *I jump or am startled by unexpected sounds*) that ranged from 0 (*never*) to 5 (*very often*) (Figley and Stamm, 1996). Cronbach’s alpha for compassion fatigue was 0.90 which was consistent with prior research where compassion fatigue had a Cronbach’s alpha value of 0.87 (Stamm, 2002; Bride et al., 2007).

#### Moral Injury

To assess moral injury, the *Moral Injury Events Scale* (MIES – Nash et al., 2013)^3^ was used. Participants completed MIES, a self-report questionnaire consisting of a Likert-type scale of 9 items ranging from 1 (strongly agree) indicating lower moral injury to 6 (strongly disagree) indicating greater moral injury (Bryan et al., 2014). Moral injury was operationalized based on the respondent’s exposure to “perceived offenses” and/or exposure to others’ “perceived betrayals” (e.g. “*I am troubled by having acted in ways that violated my own morals or values*,” “*I feel betrayed from fellow service members who I once trusted*”). In the present study, MIES demonstrated acceptable reliability with a Cronbach’s alpha score of 0.75.

#### PTSD

To assess for symptoms of PTSD in accordance with the DSM-IV criteria of PTSD symptomatology (e.g. “*repeated and disturbing memories, thoughts, or images of a stressful experience from the past*”), officers completed the *PTSD Checklist-Civilian (PCL-C –* Weathers et al., 1993). The screening questionnaire consists of 17 items based on a 5-point Likert scale (e.g. *not at all, a little bit, moderately, quite a bit, extremely*; Weathers et al., 1993). Respondents reported the extent to which each symptom had disturbed them in the last month; total scores ranged from 17 to 85 and a score greater than 50 (cut off method) signified experiencing PTSD. In the present study, PCL-C demonstrated high reliability with a Cronbach’s alpha score of 0.93, which is consistent with prior research conducted on veterans where PCL-C reliability and convergent validity with other known PTSD scales (e.g. Mississippi Scale; Keane et al., 1988; Andrykowski et al., 1998; Conybeare et al., 2012) yielded high values (e.g. PCL-C showed a coefficient alpha of 0.97 and a test-retest reliability of 0.96). Cronbach’s alpha for the three subscales were 0.89 (Re-experiencing), 0.86 (Avoidance), and 0.80 (Hyperarousal).

## Results

Two path models were tested to examine the influence of police moral injury and compassion fatigue on PTSD (first path model) and the respective PTSD clusters of re-experiencing, avoidance, and hyperarousal (second path model) using the AMOS 26 program. Since the total PTSD score and the three PTSD subscale scores were highly skewed, 95% bias-corrected confidence intervals generated by 1,000 bootstrap samples were used to assess statistical significance (Kline, 2016).

**FIGURE 1:**
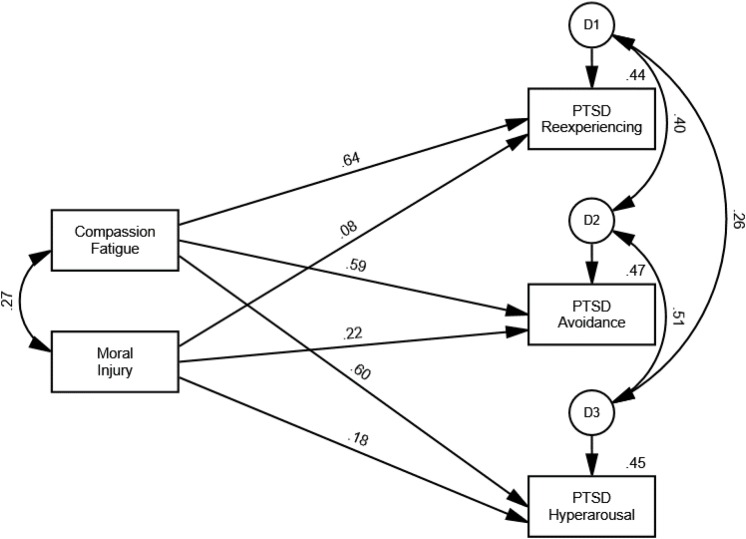
Path model for the PTSD clusters.

The results yielded by the first path model indicated that compassion fatigue (β = 0.68, *p* = 0.003) and moral injury (β = 0.20, *p* = 0.001) significantly predicted PTSD. Both predictors accounted for 57.2% of the variance of PTSD. The second path model results revealed that compassion fatigue significantly predicted all three PTSD clusters (see Table 2). Moral injury also significantly predicted re-experiencing (β = 0.08, *p* = 0.033), avoidance (β = 0.22, *p* = 0.002), and hyperarousal (β = 0.18, *p* = 0.001). The two predictors accounted for 44.1% of the variance of re-experiencing, 46.6% of the variance of avoidance, and 45.3% of the variance of hyperarousal (Figure 1).

## Discussion

The role of moral injury in predicting PTSD and its associated clusters was explored. Previous research has not studied this question with a law enforcement sample. The present results showed that moral injury, even when compassion fatigue was included in the path model, significantly predicted PTSD as well as its clusters (avoidance, hyperarousal, re-experiencing). These findings have contributed to a greater understanding of the relationship between moral injury and PTSD among law enforcement officers. In the following paragraphs, the implications of these results are discussed in relation to research, clinical, and law enforcement domains. The paper concludes with suggestions for future research, policy, and training.

### Research Implications

The present study is among the first to examine the association between PTSD symptoms and moral injury in a sample of law enforcement officers. Even when compassion fatigue was included in the model, the results of the study demonstrated that greater amounts of moral injury and compassion fatigue were both unique and significant predictors of higher overall PTSD symptoms. When examined further by the various PTSD symptom clusters, moral injury and compassion fatigue were both significant and unique predictors of the re-experiencing, hyperarousal, and avoidance of PTSD symptom clusters. It is imperative that future research examines the mechanisms in regards to the associations between those constructs. However, at this point, a possible interpretation of current research findings is that officers who suffer from moral injury experience elevated shame and guilt which may be related to PTSD symptom clusters of avoidance and re-experiencing respectively. In addition, the experience of moral conflicts and frustration (e.g. feeling betrayed by self or others) might be related to PTSD symptom cluster of hyperarousal.

The findings of the present study are consistent with those from Jinkerson and Battles (2019) who found that PMIEs, such as killing enemy soldiers, were related to and predicted PTSD symptom clusters. In addition, prior research has also shown that different types of traumatic experiences may influence the development of PTSD symptom clusters. For instance, Chopko et al. (2018) found that potentially traumatic events involving threat of harm to self were associated with PTSD hyperarousal and re-experiencing symptoms, while events involving the witnessing of harm to others were associated with the avoidance cluster symptoms in a sample of police officers.

### Clinical Implications

This study helps to further an understanding of clinical case conceptualizations by shedding light on additional risk factors faced by police officers who have been diagnosed with PTSD. Treatment interventions with this population can be optimized by ensuring that clinical assessments also take into account officers’ exposure to PMIEs, as well as their increased likelihood of developing moral injury and compassion fatigue. This study also highlights the risk of misdiagnosis and/or under-diagnosis. When focusing primarily on the symptoms associated with PTSD, a clinician treating police officers may be neglecting a significant contributing factor to their symptom profile. The present results reinforce the importance for the clinician to understand the range of comorbid conditions experienced by police officers (e.g. moral injury and compassion fatigue) and the extent to which these conditions differentially impact symptomatology. Thus, in addition to evaluating the presence of post-trauma symptoms, the clinician should also carefully assess their law enforcement patients’ exposure to acute traumatic incidents and PMIEs, as well as, the impact of their chronic exposure to trauma and morally injurious events (i.e., their level of compassion fatigue).

In addition to clinical interventions at the individual level, the present findings inform interventions at the organizational level. These initiatives should take place on two levels. First, greater awareness of the damage that can occur from exposure to PMIEs should lead police executives to ensure and promote the availability of psychological services for their officers (e.g. Blumberg et al., 2020). Secondly, law enforcement organizations should focus on some preventative measures to reduce the occurrence and intensity of some types of PMIEs. Specifically, a commitment to a people-focused leadership style and an organizational culture of wellness can help to minimize betrayal-focused PMIEs and to promote job performance, health, and compassion satisfaction.

### Law Enforcement Implications

Similar to policies and procedures regarding physical injuries, it is every police organization’s responsibility to educate their members on proactive measures regarding moral injury and PTSD starting at the recruitment level. In addition, police organizations should make a conscious effort to put safeguarding measures in place for units that may be at a higher risk of experiencing moral injury and PTSD. This can include psychological assessments before entering the unit and mandatory, annual check-ins with a mental health professional who can obtain a baseline of the police officers before they enter these units, which enables early detection of the onset of any difficulties.

Moral injury is omnipresent in many other aspects of policing. The most prevalent instance is when police officers fail, literally or from their own perspective, to protect the public. These include situations that are out of the officers’ control, however, they cannot accept that it is out of their control and often personalize the results. Such examples can be traced in Internet Child Exploitation investigators who are mandated to watch children who have been victimized. Another example is when police officers try to negotiate with suicidal people. It is during this time when a police officer may spend hours negotiating with, for instance, an armed and barricaded person trying to establish rapport.

Understanding moral injury and the potential impact of exposure to PMIEs or potentially traumatic incidents is only the first step. Police organizations are responsible in ensuring that before and after the occurrence of such events, their police officers are aware of the signs that may accompany a moral injury or represent a post-traumatic reaction.

## Conclusion

The present paper demonstrated a relationship among moral injury, compassion fatigue, and PTSD. Considering that present study sample was consisted of White European police officers, study findings should be generalized with caution to overall police populations especially in North America where police departments are diverse. Although future efforts will fine-tune these relationships, this is an important first step in developing greater understanding of the psychological challenges faced by law enforcement personnel. Police officers are exposed to a multiplicity of moral injury. They also routinely encounter the many events that can lead to compassion fatigue, which are independent of PMIEs and which do not meet the diagnostic criteria for PTSD. Likewise, most police officers never develop PTSD and many do not experience moral injury despite exposure to events which would “qualify” for the diagnoses. However, a substantial number of police officers report feelings of moral injury and compassion fatigue after chronic exposure to PMIEs and other potentially traumatic events encountered on the job. Therefore, it is imperative to continue research into how the negative impact of PMIEs and compassion fatigue can be mitigated or even prevented.

## Data Availability Statement

The datasets generated for this study will not be made publicly available because the data was collected by recruiting police officers. Any disclosure of data requires permission from participant officers’ organization (i.e., National Police of Finland) before corresponding author makes these data available by request.

## Ethics Statement

The studies involving human participants were reviewed and approved by the Research Ethics Board of the National Police of Finland and University of Toronto. The patients/participants provided their written informed consent to participate in this study.

## Author Contributions

KP developed the research idea, actively involved in the data collection and analysis, coordinated the co-authors in writing up the sections of the manuscript, authored paragraphs along the manuscript, and edited the final work. DB collaborated with KP in manuscript’s organization, authored the “Introduction” section, contributed in “Discussion” section, and edited the final manuscript. VC was involved in additional statistical analyses, authored parts of the “Introduction” and “Discussion” sections, also authored most of the “Results” section, and collaborated with KP in planning and running additional statistical analyses that were necessary in this study. BT analyzed the data and authored the “Results” section under the guidance of KP. KK co-authored the “Introduction” section and contributed in the organization of the manuscript. BC authored the research implications of the “Discussion” section. BM authored the law enforcement practice implications of the “Discussion” section. PA authored the “Methods” section with the guidance of KP, collaborated with BT to develop the tables, and finalized the APA-style formatting under the directions of KP. MK coordinated the data collection and recruited participants from the National Police of Finland.

## Conflict of Interest

VC is the owner of the company MV Stats Consultants. The remaining authors declare that the research was conducted in the absence of any commercial or financial relationships that could be construed as a potential conflict of interest.

## Footnotes

1 Methods of present manuscript (including results of the Missing Values Analysis) are part of first author’s dissertation work (Papazoglou, 2017).

3 Item #6 of the MIES scale was accidentally not included in the data collection survey questionnaire.
